# ﻿*Pleocatenatachiangraiensis* gen. et. sp. nov. (Pleosporales, Dothideomycetes) from medicinal plants in northern Thailand

**DOI:** 10.3897/mycokeys.87.79433

**Published:** 2022-02-11

**Authors:** Ya-Ru Sun, Ning-Guo Liu, Kevin D. Hyde, Ruvishika S. Jayawardena, Yong Wang

**Affiliations:** 1 Department of Plant Pathology, College of Agriculture, Guizhou University, Guiyang 550025, China; 2 Center of Excellence in Fungal Research, Mae Fah Luang University, Chiang Rai 57100, Thailand; 3 School of Science, Mae Fah Luang University, Chiang Rai 57100, Thailand; 4 Innovative Institute of Plant Health, Zhongkai University of Agriculture and Engineering, Haizhu District, Guangzhou 510000, China; 5 School of Life Science and Technology, Center for Informational Biology, University of Electronic Science and Technology of China, Chengdu 611731, China

**Keywords:** Genera *incertae sedis*, hyphomycetes, multi-gene phylogeny, taxonomy

## Abstract

*Pleocatenata*, a new genus, is introduced with its type species, *Pleocatenatachiangraiensis*, which was isolated from withered twigs of two medicinal plants, *Clerodendrumquadriloculare* (Blanco) Merr (Verbenaceae) and *Tarennastellulata* (Hook.f.) Ridl (Rubiaceae) in northern Thailand. The genus is characterized by mononematous, septate, brown or dark brown conidiophores, monotretic conidiogenous cells and catenate, obclavate, olivaceous to blackish brown conidia. Phylogenetic analysis of combined LSU, SSU, *tef1*-α, *rpb2* and ITS sequence data showed *Pleocatenata* forms a distinct phylogenetic lineage in Pleosporales, Dothideomycetes. Therefore, we treat *Pleocatenata* as Pleosporales genera *incertae sedis* based on morphology and phylogenetic analyses. Descriptions and illustrations of the new taxa are provided, and it is compared with morphologically similar genera.

## ﻿Introduction

Medicinal plants are a rich source of natural products with biological and chemical properties. They are used in health care or treatment of human ailments and have been used since prehistoric times worldwide (Rasool-Hassan 2012). Many fungi have been found on medicinal plants and are members of Dothideomycetes and Sordariomycetes (Bhagat et al. 2012; Long et al. 2019; Ma et al. 2019; Hyde et al. 2020; Tennakoon et al. 2021). They form important associations with medicinal plants and as pathogens or saprobes (Long et al. 2019; Tennakoon et al. 2021), sources of medicines (Strobel et al. 1993; Huang et al. 2008; Hyde et al. 2019), involved in nutrient recycling (Bonnardeaux et al. 2007) and some are used in biological control (Hyde et al. 2019).

Pleosporales is the largest order in Dothideomycetes, which accounts for about a quarter of the class (Zhang et al. 2012; Hyde et al. 2013; Hongsanan et al. 2020a). They have a worldwide distribution with diverse lifestyles, including saprobes, pathogens of plants and humans, endophytes, epiphytes and hyperparasites (Ramesh 2003; Kirk et al. 2008; Zhang et al. 2012; Hyde et al. 2013; Sun et al. 2019; Ferdinandez et al. 2021). Many species in *Alternaria* Nee*s*, *Curvularia* Boedijn and *Corynespora* Güssow, can invade medicinal plants and cause leaf spots and other diseases, as economically important plant pathogens (Mathiyazhagan et al. 2004; Abtahi and Nourani 2017; Zhang et al. 2020), and some also pose a threat to human health (Hyde et al. 2018; Iturrieta-González et al. 2020). Endophytes in Pleosporales also show important biocontrol value (Su et al. 2014; De Silva et al. 2019; Hyde et al. 2019), for example, an extract from *Cochliobolusspicifer* R.R. Nelson has mosquito-larvicidal activity (Abutaha et al. 2015).

The sexual morph of Pleosporales is characterized by uniloculate ascomata typically with papillae, ostioles and pseudoparaphyses, generally fissitunicate asci bearing mostly septate ascospores of different colours and shapes (Ramesh 2003; Kirk et al. 2008; Zhang et al. 2012; Hyde et al. 2013). Coelomycetes and hyphomycetes are the asexual morphs of pleosporalean taxa (Zhang et al. 2012; Hongsanan et al. 2020a). Recent comprehensive studies on Dothideomycetes treated 91 families in Pleosporales (Hongsanan et al. 2020a). More than 40 genera are recognized as genera *incertae sedis* in Pleosporales (Hongsanan et al. 2020a; Wijayawardene et al. 2020, 2021). This uncertainty in genetic placement occurs for the following reasons: 1) some genera lack sufficient collections even though molecular data is available, they are not included in any families in phylogenetic analyses, eg. *Aegeanispora* E.B.G. Jones & Abdel-Wahab, *Antealophiotrema* A. Hashim. & Kaz. Tanaka and *Perthomyces* Crous (Li et al. 2016; Abdel-Wahab et al. 2017; Crous et al. 2017); 2) due to the diverse morphology of hyphomycetous asexual morphs, it is difficult to determine their familial placement without the sexual morph and molecular data. Examples are *Briansuttonia* R.F. Castañeda, Minter & Saikawa, *Cheiromoniliophora* Tzean & J.L. Chen, *Dangeardiella* Sacc. & P. Syd and *Pleosphaerellula* Naumov & Czerepan (Obrist 1959; Tóth 1975; Tzean and Chen 1990; Castañeda-Ruiz et al. 2004).

During the examination of collections from medicinal plants in northern Thailand (Sun et al. 2021), two isolates representing a new species were obtained from *Clerodendrumquadriloculare* and *Tarennastellulata*. Morphology and phylogenetic analyses confirmed that it was distinct in Pleosporales, but its familial placement was uncertain. Thus, we introduced a new genus, *Pleocatenata* (Pleosporales, genera *incertae sedis*) to accommodate the new species, *P.chiangraiensis*.

## ﻿Materials and methods

### ﻿Collection, examination and isolation

The isolates used in this study were collected from decaying twigs of *Clerodendrumquadriloculare* and *Tarennastellulata* from Mae Fah Luang University, Chiang Rai, Thailand during June to July 2020 in terrestrial habitat. The samples were packaged in envelopes and returned to the laboratory as described in Senanayake et al. (2020). The fruiting bodies on natural substrates were observed and photographed using a stereo-microscope (SteREO Discovery, V12, Carl Zeiss Microscopy GmBH, Germany). Morphological characters were observed using a Nikon ECLIPSE Ni compound microscope (Nikon, Japan) and photographed with a Nikon DS-Ri2 digital camera (Nikon, Japan). The Adobe Photoshop CS6 Extended v. 13.0 software was used to make photo-plates. Measurements were done with the Tarosoft (R) Image Frame Work software.

Single spore isolations were used to obtain pure cultures following the methods described by Senanayake et al. (2020). Germinated conidia were transferred to new potato dextrose agar (PDA) plates and incubated at 26 °C for four weeks. The pure cultures obtained were deposited in Mae Fah Luang University Culture Collection (MFLUCC), Chiang Rai, Thailand. Herbaria materials were deposited in the herbarium of Mae Fah Luang University (MFLU), Chiang Rai, Thailand. Facesoffungi (FoF) and Index Fungorum numbers were acquired as described in Jayasiri et al. (2015) and Index Fungorum (2022).

### ﻿DNA extraction, PCR amplification and sequencing

Fresh fungal mycelia grown on PDA medium for 4 weeks at 26 °C were scraped with a sterile scalpel. Genomic DNA was extracted from scraped mycelia using the BIOMIGA Fungus Genomic DNA Extraction Kit (GD2416, BIOMIGA, San Diego, California, USA) following the manufacture’s protocol. Five genes were selected in this study: the 28S subunit rDNA (LSU), the 18S subunit rDNA (SSU), the internal transcribed spacers (ITS), the translation elongation factor 1 (*tef1-α*), and the RNA polymerase II subunit 2 (*rpb2*). Polymerase chain reaction (PCR) was carried out in 20 μL reaction volume which contained 10 μL 2 × PCR Master Mix, 7 μL ddH_2_O, 1 μL of each primer, and 1 μL template DNA. The PCR thermal cycle program and primers are given (Table 1). Purification and sequencing of PCR products were carried out at SinoGenoMax (Beijing) Co., China.

**Table 1.** Primers and PCR procedures used in this study.

Locus	Primers		PCR procedures	References
	Name	Sequence (5’–3’)		
Large subunit (LSU)	LR0R	ACCCGCTGAACTTAAGC	94 °C 3 min; 35 cycles of 94 °C 30 s, 52 °C 30 s, 72 °C 1 min; 72 °C 8 min; 4 °C on hold	Vilgalys and Hester (1990), Rehner and Samuels (1994)
	LR5	TCCTGAGGGAAACTTCG		
Small subunit (SSU)	NS1	GTAGTCATATGCTTGTCTC		White et al. (1990)
	NS4	CTTCCGTCAATTCCTTTAAG		
Internal transcribed spacer (ITS)	ITS5	GGAAGTAAAAGTCGTAACAAGG		
	ITS4	TCCTCCGCTTATTGATATGC		
Elongation factor-1 alpha (*tef1*-α)	EF1-983F	GCYCCYGGHCAYCGTGAYTTYAT	94 °C 2 min; 36 cycles of 66 °C – 56 °C (touchdown 9 cycles), 94 °C 30 sec, 56 °C 1 min, 72 °C 1 min; 72 °C 10 min; 4 °C on hold	Rehner and Buckley (2005)
	EF1-2218R	ATGACACCRACRGCRACRGTYTG		
RNA polymerase II subunit (*rpb2*)	fRPB2-5F	GAYGAYMGWGATCAYTTYGG	94 °C 3 min; 40 cycles of 94 °C 20 sec, 55 °C 30 sec, 72 °C 1 min; 72 °C 10 min; 4 °C on hold	Liu et al. 1999
	fRPB2-7cR	CCCATRGCTTGYTTRCCCAT		

### ﻿Phylogenetic analyses

BLASTn (https://blast.ncbi.nlm.nih.gov//Blast.cgi) was used to evaluate closely related strains to our new taxa. Other sequences used in this study were obtained from GenBank referring to Zhang et al. (2012, 2018) and Hongsanan et al. (2020a, 2021) (Table 2). The single gene sequences were viewed using BioEdit v. 7.0.9.0 (Hall 1999). Alignments for each locus were generated with MAFFT v.7 (https://mafft.cbrc.jp/alignment/server/) and manually improved using AliView (Larsson 2014) for maximum alignment and minimum gaps. The final single gene alignments were combined by SequenceMatrix 1.7.8 (Vaidya et al. 2011).

**Table 2.** Taxa of Pleosporales used in the phylogenetic analysis with the corresponding GenBank accession numbers. The newly generated strains are indicated in bold. N/A: Not available.

Species names	Strain number	LSU	SSU	ITS	*tef1*-α	*rpb2*
*Acrocalymmaaquatica*	MFLUCC 11-0208	JX276952	JX276953	JX276951	N/A	N/A
*Acrocalymmapterocarpi*	MFLUCC 17-0926	MK347949	MK347840	MK347732	MK360040	N/A
*Acuminatisporapalmarum*	MFLUCC 18-0264	MH390437	MH390401	NR_163327	MH399248	N/A
	MFLUCC 18-0460	MH390438	MH390402	MN749106	MH399249	N/A
*Aigialusgrandis*	BCC 20000	GU479775	GU479739	N/A	GU479839	N/A
*Alternariaalternata*	AFTOL ID-1610	DQ678082	KC584507	KF465761	KC584634	KC584375
*Amniculicolaaquatica*	MFLUCC 16-1123	MK106096	MK106108	N/A	MK109800	N/A
*Amorocoelophomacassia*	MFLUCC 17-2283	MK347956	NG_065775	MK347739	MK360041	MK434894
*Angustimassarinalonicerae*	MFLUCC 15-0087	KY496724	N/A	KY496759	N/A	N/A
*Anteagloniumparvulum*	SMH5223	GQ221909	N/A	N/A	GQ221918	N/A
*Aquasubmersajaponica*	HHUF 30469	NG_057138	NG_062426	NR_154739	LC194384	LC194421
*Aquasubmersamircensis*	MFLUCC 11-0401	NG_042699	NG_061141	JX276954	N/A	N/A
*Ascocylindricamarina*	MD6011	KT252905	KT252907	N/A	N/A	N/A
	MF416	MK007123	MK007124	N/A	N/A	N/A
*Astragalicolavasilyevae*	MFLUCC 17-0832	MG828986	MG829098	NR_157504	MG829193	MG829248
*Astrosphaeriellafusispora*	MFLUCC 10-0555	KT955462	KT955443	N/A	KT955425	KT955413
*Atrocalyxacutisporus*	KT 2436	LC194341	LC194299	LC194475	LC194386	LC194423
*Bahusandhikaindica*	GUFCC 18001	KF460274	N/A	KF460273	N/A	N/A
*Bambusicolabambusae*	MFLUCC 11-0614	JX442035	JX442039	JX442031	N/A	KP761718
*Berkleasmiumcrunisia*	BCC 17023	DQ280271	N/A	DQ280265	N/A	N/A
*Berkleasmiumtyphae*	BCC 12536	DQ280275	N/A	DQ280264	N/A	N/A
*Brevicollumhyalosporum*	MFLUCC 17-0071	MG602200	MG602202	MG602204	MG739516	N/A
*Brevicollumversicolor*	HHUF 30591	NG_058716	NG_065124	NR_156335	LC271246	LC271250
*Camarosporidiellacaraganicola*	MFLUCCC 14-0605	KP711381	KP711382	KP711380	N/A	N/A
*Camarosporiumquaternatum*	CPC 31081	NG_064442	KY929123	NR_159756	KY929201	N/A
*Camarosporomycesflavigenus*	CBS 314.80	GU238076	NG_061093	MH861266	N/A	N/A
*Coniothyriumpalmarum*	CBS 400.71	JX681084	EU754054	MH860184	N/A	KT389592
*Corynesporacassiicola*	CBS 100822	GU301808	GU296144	N/A	GU349052	GU371742
*Corynesporatorulosa*	CPC 15989	KF777207	N/A	NR_145181	N/A	N/A
*Crassiperidiumoctosporum*	MAFF 246406	LC373116	LC373092	LC373104	LC373128	LC373140
*Cryptocoryneumjaponicum*	HHUF 30482	NG_059035	NG_065118	NR_153938	LC096144	LC194438
*Cryptocoryneumpseudorilstonei*	CBS 113641	NG_059036	LC194322	NR_153941	LC096152	LC194446
*Cucurbitariaberberidis*	MFLUCC 11-0387	KC506796	KC506800	N/A	N/A	N/A
*Cyclothyriellarubronotata*	CBS 141486	KX650544	NG_061252	NR_147651	KX650519	KX650574
*Cylindroaseptosporaleucaenicola*	MFLUCC 17-2424	MK347966	MK347856	NR_163333	MK360047	N/A
*Dacampiaengeliana*	Hafellner 72868	KT383791	N/A	N/A	N/A	N/A
*Dacampiahookeri*	Hafellner 73897	KT383792	N/A	N/A	N/A	N/A
*Delitschiachaetomioides*	SMH 3253.2	GU390656	N/A	N/A	GU327753	N/A
*Delitschiawinteri*	AFTOL ID-1599	DQ678077	DQ678026	N/A	DQ677922	DQ677975
*Dendryphionfluminicola*	MFLUCC 17-1689	MG208141	N/A	NR_157490	MG207992	N/A
*Dictyocheirosporabannica*	KH 332	AB807513	AB797223	LC014543	AB808489	N/A
*Dictyosporiumelegans*	NBRC 32502	DQ018100	DQ018079	DQ018087	N/A	N/A
*Didymellaexigua*	CBS 183.55	MH868977	GU296147	MH857436	N/A	N/A
*Didymellarumicicola*	CBS 683.79	MH873007	N/A	KT389503	N/A	KT389622
*Didymosphaeriarubi-ulmifolii*	MFLUCC 14-0023	KJ436586	KJ436588	MK646049	N/A	N/A
*Dimorphosporicolatragani*	CBS 570.85	KU728536	N/A	KU728497	N/A	N/A
*Dothidotthiaaceris*	MFLUCC 16-1183	MK751816	MK751761	MK751726	N/A	N/A
*Fissuromacalami*	MFLUCC 13-0836	MF588993	NG_062430	N/A	MF588975	N/A
*Flammeascomabambusae*	MFLU 11-0143	NG_059553	KP753952	NR_132915	N/A	N/A
*Flavomycesfulophazii*	CBS 135761	NG_058131	NG_061191	NR_137960	N/A	N/A
*Foliophomafallens*	CBS 161.78	GU238074	GU238215	KY940772	N/A	KC584502
	CBS 284.70	GU238078	GU238218	MH859609	N/A	N/A
*Fuscostagonosporacytisi*	MFLUCC 16-0622	KY770978	KY770977	N/A	KY770979	N/A
*Fuscostagonosporasasae*	HHUF 29106	AB807548	AB797258	AB809636	AB808524	N/A
*Fusculinaeucalypti*	CBS 120083	DQ923531	N/A	DQ923531	N/A	N/A
*Fusculinaeucalyptorum*	CBS 145083	MK047499	N/A	NR_161140	N/A	N/A
*Halojulellaavicenniae*	BCC 20173	GU371822	GU371830	N/A	GU371815	GU371786
*Halotthiaposidoniae*	BBH 22481	GU479786	GU479752	N/A	N/A	N/A
*Hazslinszkyomycesaloes*	CBS 136437	KF777198	N/A	KF777142	N/A	N/A
*Helminthosporiumvelutinum*	L131	KY984352	KY984432	KY984352	KY984463	KY984413
*Hermatomycesiriomotensis*	HHUF 30518	LC194367	LC194325	LC194483	LC194394	LC194449
*Hermatomycestectonae*	MFLUCC 14-1140	KU764695	KU712465	KU144917	KU872757	KU712486
*Hypsostromacaimitalense*	GKM1165	GU385180	N/A	N/A	N/A	N/A
*Hypsostromasaxicola*	SMH5005	GU385181	N/A	N/A	N/A	N/A
*Hysteriumangustatum*	CBS 123334	FJ161207	N/A	N/A	N/A	N/A
*Hysterobreviumsmilacis*	CBS 114601	FJ161174	FJ161135	N/A	FJ161091	FJ161114
*Latoruacaligans*	CBS 576.65	NG_058180	N/A	N/A	N/A	N/A
*Latoruagrootfonteinensis*	CBS 369.72	NG_058181	N/A	N/A	N/A	N/A
*Lentimurisporaurniformis*	MFLUCC 18-0497	MH179144	MH179160	N/A	MH188055	N/A
*Lentitheciumclioninum*	HHUF 28199	NG_059391	NG_064845	NR_154137	AB808515	N/A
*Lentitheciumpseudoclioninum*	HHUF 29055	NG_059392	NG_064847	AB809633	AB808521	N/A
*Lepidosphaerianicotiae*	AFTOL ID-1576	DQ678067	N/A	N/A	DQ677910	DQ677963
*Leptosphaeriacichorium*	MFLUCC 14-1063	KT454712	KT454728	KT454720	N/A	N/A
*Leucaenicolaphraeana*	MFLUCC 18-0472	MK348003	NG_065784	MK347785	MK360060	MK434867
*Libertasomycesmyopori*	CPC 27354	NG_058241	N/A	KX228281	N/A	N/A
*Ligninsphaeriajonesii*	MFLUCC 15-0641	NG_059642	N/A	N/A	N/A	N/A
*Lindgomycescigarospora*	G619	KX655804	KX655805	KX655794	N/A	N/A
*Lindgomycesingoldianus*	ATCC 200398	AB521736	NG_016531	NR_119938	N/A	N/A
*Longiostiolumtectonae*	MFLUCC 12-0562	KU764700	N/A	KU712447	N/A	N/A
*Longipedicellataaptrootii*	MFLU 10-0297	KU238894	KU238895	KU238893	KU238892	KU238891
*Lophiostomamacrostomum*	KT508	AB619010	AB618691	N/A	LC001751	N/A
*Lophiotremaeburnoides*	KT 1424.1	LC001707	LC001706	LC001709	LC194403	LC194458
*Macrodiplodiopsisdesmazieri*	CBS 140062	NG_058182	N/A	NR_132924	N/A	N/A
*Massariaanomia*	CBS 59178	GU301839	GU296169	N/A	N/A	GU371769
*Massariainquinans*	M19	N/A	HQ599444	HQ599402	HQ599342	HQ599460
*Melanommajaponicum*	MAFF 239634	NG_060360	NG_065122	NR_154215	LC203367	LC203395
*Melanommapulvispyrius*	CBS 124080	MH874873	GU456302	MH863349	GU456265	GU456350
*Misturatosphaeriaaurantonotata*	GKM 1238	NG_059927	N/A	N/A	GU327761	N/A
*Morosphaeriamuthupetensis*	NFCCI4219	MF614796	MF614797	MF614795	MF614798	N/A
*Morosphaeriavelatispora*	KH221	AB807556	AB797266	LC014572	AB808532	N/A
*Multiloculariabambusae*	MFLUCC 11-0180	KU693438	KU693442	KU693446	N/A	N/A
*Murisporagalii*	MFLUCC 13-0819	KT709175	KT709182	KT736081	KT709189	N/A
*Neocamarosporiumgoegapense*	CPC 23676	KJ869220	N/A	KJ869163	N/A	N/A
*Neoconiothyriumpersooniae*	CBS 143175	MG386094	N/A	MG386041	N/A	N/A
*Neomassariafabacearum*	MFLUCC 16-1875	KX524145	NG_061245	N/A	KX524149	N/A
*Neomassariaformosana*	NTUCC 17-007	MH714756	MH714759	N/A	MH714762	MH714765
*Neomassarinathailandica*	MFLU 11-0144	NG_059718	N/A	NR154244	N/A	N/A
	MFLUCC 17-1432	MT214467	MT214420	MT214373	N/A	N/A
*Neopaucisporarosaecae*	MFLUCC 17-0807	MG829033	NG_061293	MG828924	MG829217	N/A
*Neophaeosphaeriaagaves*	CPC 21264	KF777227	N/A	KF777174	N/A	N/A
*Neophaeosphaeriafilamentosa*	CBS 102202	GQ387577	GQ387516	JF740259	GU349084	GU371773
*Neophaeosphaeriaphragmiticola*	KUMCC 16-0216	MG837009	NG_065735	N/A	MG838020	N/A
*Neoplatysporoidesaloes*	CPC 36068	MN567619	N/A	NR_166316	N/A	N/A
*Neopyrenochaetacercidis*	MFLUCC 18-2089	MK347932	MK347823	MK347718	N/A	MK434908
*Neopyrenochaetopsishominis*	UTHSC DI16 238	LN907381	N/A	LT592923	N/A	LT593061
*Neoroussoellabambusae*	MFLUCC 11-0124	KJ474839	N/A	KJ474827	KJ474848	KJ474856
*Neotestudinarosatii*	CBS 690.82	DQ384107	DQ384069	N/A	N/A	N/A
*Neoyrenochaetaacicola*	CBS 812.95	GQ387602	GQ387541	NR_160055	N/A	LT623271
*Nigrogranafuscidula*	CBS 141556	KX650550	N/A	NR_147653	KX650525	N/A
*Nigrogranamackinnonii*	CBS 674.75	GQ387613	NG_061081	NR_132037	KF407986	KF015703
*Occultibambusabambusae*	MFLUCC 13-0855	KU863112	N/A	KU940123	KU940193	KU940170
*Occultibambusajonesii*	GZCC 16-0117	KY628322	KY628324	N/A	KY814756	KY814758
*Parabambusicolabambusina*	KH 139	AB807537	AB797247	LC014579	AB808512	N/A
*Paradictyoarthriniumaquatica*	MFLUCC 16-1116	NG_064501	N/A	NR_158861	N/A	N/A
*Paradictyoarthriniumdiffractum*	MFLUCC 13-0466	KP744498	KP753960	KP744455	N/A	KX437764
*Paralophiostomahysterioides*	PUFNI 17617	MT912850	MN582762	MN582758	N/A	MT926117
*Parapyrenochaetaprotearum*	CBS 131315	JQ044453	N/A	JQ044434	N/A	LT717683
*Periconiadelonicis*	MFLUCC 17-2584	NG_068611	NG_065770	N/A	N/A	MK434901
*Periconiapseudodigitata*	KT 1395	AB807564	AB797274	LC014591	N/A	N/A
*Phaeoseptummali*	MFLUCC 17-2108	MK625197	N/A	MK659580	MK647990	MK647991
*Phaeoseptumterricola*	MFLUCC 10-0102	MH105779	MH105780	MH105778	MH105781	MH105782
*Phaeosphaeriaoryzae*	CBS 110110	KF251689	GQ387530	KF251186	N/A	KF252193
*Phaeosphaeriopsistriseptata*	MFLUCC 13-0271	KJ522479	KJ522484	KJ522475	MG520919	KJ522485
*Plenodomussalvia*	MFLUCC 13-0219	KT454717	KT454732	KT454725	N/A	N/A
***Pleocatenatiumchiangraiense***	**MFLUCC 21-0222**	**OL986398**	**N/A**	**OL986396**	**OM240638**	**OM117709**
	**MFLUCC 21-0223**	**OL986399**	**N/A**	**OL986397**	**OM240637**	**OM117708**
*Pleohelicoonrichonis*	CBS 282.54	N/A	AY856952	MH857332	N/A	N/A
*Pleomonodictysdescalsii*	FMR 12716	KY853522	N/A	KY853461	N/A	N/A
*Preussiafuniculate*	CBS 659.74	GU301864	GU296187	N/A	GU349032	GU371799
*Pseudoastrosphaeriellalongicolla*	MFLUCC 11-0171	KT955476	N/A	N/A	KT955438	KT955420
*Pseudoastrosphaeriellathailandensis*	MFLUCC 11-0144	KT955478	KT955457	N/A	KT955440	KT955416
*Pseudoberkleasmiumchiangmaiense*	MFLUCC 17-1809	MK131260	N/A	MK131259	MK131261	N/A
*Pseudoberkleasmiumpandanicola*	KUMCC 17-0178	MH260304	MH260344	MH275071	N/A	N/A
*Pseudocoleodictyosporatectonae*	MFLUCC 12-0385	KU764709	NG_061232	NR_154338	N/A	KU712491
*Pseudocoleodictyosporathailandica*	MFLUCC 12-0565	KU764701	NG_062417	NR_154337	N/A	KU712494
*Pseudodidymosphaeriaspartii*	MFLUCC 13-0273	KP325436	KP325438	KP325434	N/A	N/A
*Pseudopyrenochaetalycopersici*	FMR 15746	EU754205	NG_062728	NR_103581	N/A	LT717680
*Pseudopyrenochaetaterretris*	FMR 15327	LT623216	N/A	LT623228	N/A	LT623287
*Pseudotetraploalongissima*	HC 4933	AB524612	AB524471	AB524796	AB524827	N/A
*Pseudoxylomyceselegans*	KT 2887	AB807598	AB797308	LC014593	AB808576	N/A
*Pyrenochaetopsisleptospora*	CBS 101635	GQ387627	NG_063097	JF740262	MF795881	LT623282
*Pyrenochaetopsistabarestanensis*	IBRC M 30051	KF803343	NG_065034	NR_155636	N/A	N/A
*Quadricrurabicornis*	yone 153	AB524613	AB524472	AB524797	AB524828	N/A
*Quercicolafusiformis*	MFLUCC 18-0479	MK348009	MK347898	MK347790	MK360085	MK434864
*Quercicolaguttulospora*	MFLUCC 18-0481	MK348010	MK347899	MK347791	MK360086	N/A
*Quixadomycescearensis*	HUEFS 238438	MG970695	N/A	NR_160606	N/A	N/A
*Roussoellanitidula*	MFLUCC 11-0634	KJ474842	N/A	KJ474834	KJ474851	KJ474858
*Salsugineaphoenicis*	MFLU 19-0015	MK405280	N/A	N/A	MK404650	N/A
*Salsuginearamicola*	KT 2597.2	GU479801	GU479768	N/A	GU479862	GU479834
*Seltsamiaulmi*	CBS 143002	MF795794	MF795794	MF795794	MF795882	MF795836
*Shiraiabambusicola*	GZAAS2.629	KC460980	N/A	GQ845415	N/A	N/A
*Splanchnonemaplatani*	CBS 222.37	KR909316	KR909318	MH855895	KR909319	KR909322
*Sporormiafimetaria*	UPS Dissing Gr.81.194	GQ203729	N/A	GQ203769	N/A	N/A
*Sporormiellaisomera*	CBS 166.73	MH872355	N/A	AY943053	N/A	N/A
*Stemphyliumherbarum*	CBS 191.86	GU238160	GU238232	NR_111243	KC584731	DQ247794
*Striatiguttulanypae*	MFLUCC 18-0265	MK035992	MK035977	MK035969	MK034432	MK034440
*Striatiguttulaphoenicis*	MFLUCC 18-0266	MK035995	MK035980	MK035972	MK034435	MK034442
*Sublophiostomathailandica*	MFLUCC 11-0185	KX534216	KX534222	MW136275	KX550080	MW088718
	MFLUCC 11-0207	KX534212	KX534218	MW136257	KX550077	MW088714
*Subplenodomusviolicola*	CBS 306.68	MH870849	GU238231	MH859138	N/A	N/A
*Sulcatisporaacerina*	KT 2982	LC014610	LC014605	LC014597	LC014615	N/A
*Sulcatisporaberchemiae*	KT 1607	AB807534	AB797244	AB809635	AB808509	N/A
*Sulcosporiumthailandica*	MFLUCC 12-0004	KT426563	KT426564	MG520958	N/A	N/A
*Teichosporatrabicola*	C134	KU601591	N/A	KU601591	KU601601	KU601600
*Tetraplosphaeriasasicola*	KT 563	AB524631	AB524490	AB524807	AB524838	N/A
*Thyridariaacaciae*	CBS 138873	NG_058127	N/A	KP004469	N/A	N/A
*Thyridariabroussonetiae*	TB1	KX650568	KX650515	KX650568	KX650539	KX650586
*Torulaaquatica*	MFLUCC 16-1115	MG208146	N/A	MG208167	N/A	MG207977
*Torulapluriseptata*	MFLUCC 14-0437	KY197855	KY197862	MN061338	KY197875	KY197869
*Tremateiaarundicola*	MFLU 16-1275	KX274248	KX274254	KX274241	KX284706	N/A
*Trematosphaeriagrisea*	CBS 332.50	NG_057979	NG_062930	NR_132039	KF015698	KF015720
*Trematosphaeriapertusa*	CBS 122368	NG_057809	FJ201991	NR_132040	KF015701	FJ795476
*Tzeananiataiwanensis*	NTUCC 17-006	MH461121	MH461127	MH461124	MH461131	N/A
*Wicklowiaaquatica*	CBS 125634	MH875044	NG_061099	N/A	N/A	N/A
*Wicklowiasubmersa*	MFLUCC 18-0373	MK637644	MK637643	N/A	N/A	N/A
*Xenopyrenochaetopsispratorum*	CBS 445.81	GU238136	NG_062792	MH861363	N/A	KT389671

The single locus and combined analyses were carried out for maximum likelihood (ML) and Bayesian posterior probability (BYPP). The ML analyses were carried out using IQ-TREE (Nguyen et al. 2015; Trifinopoulos et al. 2016) on the IQ-TREE web server (http://iqtree.cibiv.univie.ac.at, 30 September 2021) under partitioned models. The best-fit substitution models were determined by WIQ-TREE (Chernomor et al. 2016): SYM+I+G4 for LSU and SSU; TIM+F+I+G4 for *tef1*-α; GTR+F+I+G4 for *rpb2*; TIM2+F+I+G4 for ITS. Ultrafast bootstrap analysis was implemented with 1,000 replicates (Minh et al. 2013; Hoang et al. 2018).

The BYPP analyses were performed in CIPRES (Miller et al. 2010) with MrBayes on XSEDE 3.2.7a (Ronquist et al. 2012). The best nucleotide substitution model for each data partition was evaluated by MrModeltest 2.2 (Nylander 2004). The substitution model GTR+I+G was decided for LSU, SSU, ITS, *tef1*-α and *rpb2* sequences. The Markov chain Monte Carlo (MCMC) sampling approach was used to calculate posterior probabilities (PP) (Rannala and Yang 1996). Six simultaneous Markov chains were run for 10 million generations and trees were sampled every 1,000^th^ generation. The first 20% of trees, representing the burn-in phase of the analyses, were discarded and the remaining trees were used for calculating posterior probabilities (PP) in the majority rule consensus tree.

Phylogenetic trees were viewed using FigTree v1.4.0 (Rambaut and Drummond 2008) and modified in Microsoft Office PowerPoint 2010 and converted to jpg file using Adobe Photoshop CS6 Extended 10.0 (Adobe Systems, San Jose, CA, USA). The new sequences derived from this study were deposited in GenBank. The final alignment and tree were deposited in TreeBase (http://purl.org/phylo/treebase/phylows/study/TB2:S29199).

## ﻿Results

### ﻿Phylogenetic analyses

**Figure 1.**
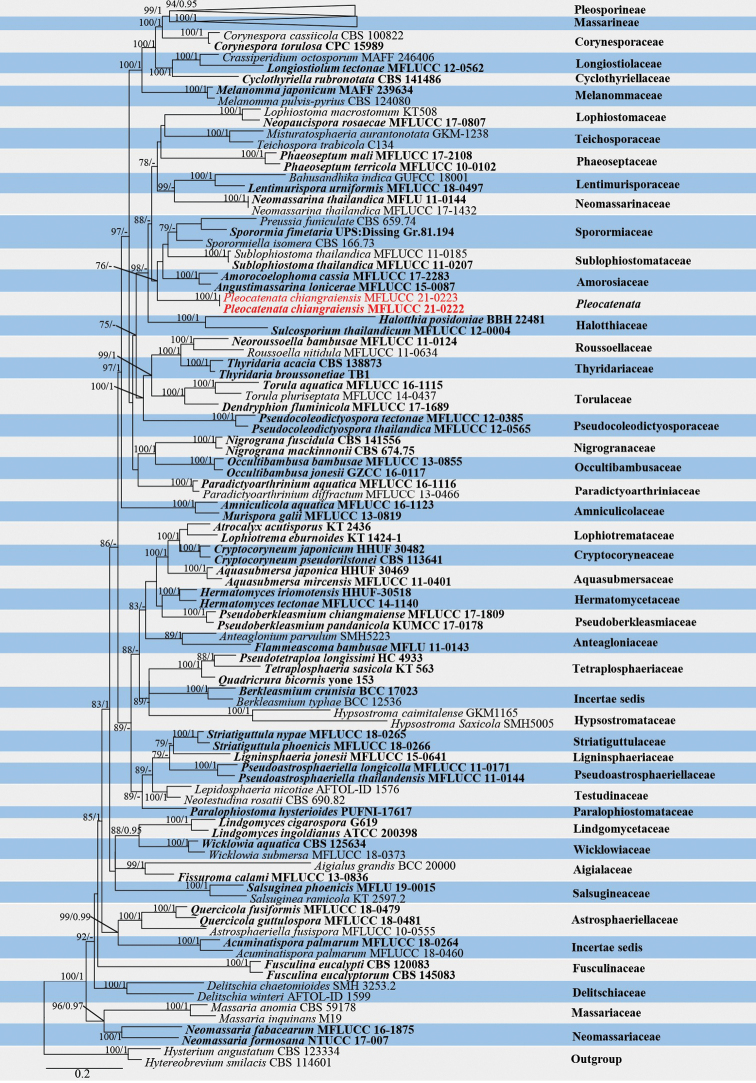
Maximum likelihood tree generated by IQ-Tree, based on analysis of a combined dataset of LSU, SSU, *tef1*-α, *rpb2* and ITS sequence data. Bootstrap support values for ML greater than 75% and Bayesian posterior probabilities greater than 0.95 are given near nodes, respectively. Ex-type strains are in bold, the new isolates are in red.

Blast searches of LSU, *tef1*-α, *rpb2* and ITS sequences data in NCBI showed that our sequences were related to Acrocalymmaceae, Amorosiaceae, Sporormiaceae and Sublophiostomataceae. One hundred and seventy-six taxa, representing all families in Pleosporales, with *Hysteriumangustatum* Alb. & Schwein (CBS 123334) and *Hysterobreviumsmilacis* (Schwein.) E. Boehm & C.L. Schoch (CBS 114601) as the outgroups, were selected for the analyses. The final combined dataset consisted of 4,953 characters (LSU: 1–850 bp, SSU: 851–1,851 bp, *tef1*-α: 1,852–2,720 bp, *rpb2*: 2,721–3,701 bp, ITS: 3,702–4,953 bp), including alignment gaps. Among them, 2,336 characters were constant, 608 variable characters were parsimony-uninformative, and 2,009 characters were parsimony informative. The most likely tree (-ln = 98,965.704) is presented (Figure. 1) to show the phylogenetic placement of the newly introduced genus and its relationship with other members in Pleosporales.

Analyses of both ML and BYPP (not shown) yielded almost identical results, and the topology of the trees were similar to previous studies (Zhang et al. 2018; Hongsanan et al. 2020a, 2021). The combined analyses showed that two suborders Massarineae and Pleosporineae were well-supported and formed an upper clade in Pleosporales. Our two newly obtained fungal isolates (MFLUCC 21-0222 and MFLUCC 21-0223) clustered together and formed a distinct clade with maximum support (ML-BS = 100%, BYPP = 1.00) and they grouped with Amorosiaceae, Sporormiaceae and Sublophiostomataceae with weak support.

### ﻿Taxonomy

#### ﻿ Pleocatenata

Taxon classificationFungiPleosporalesHyphomycetous

Y.R. Sun, Yong Wang bis & K.D. Hyde gen. nov.

402C64E6-8895-5501-81E8-069D0B58EF71

Index Fungorum number:IF559457

Facesoffungi number: FoF 10630

##### Etymology.

“Pleo-” an abbreviation of Pleosporales, the order in which this fungus is classified; “-*catenata*” refers to the catenate conidia of this fungus.

##### Description.

*Saprobic* on decaying twigs in terrestrial habitats. **Asexual morph**: Hyphomycetous. *Colonies* on natural substrate effuse, dark, velvety. *Conidiophores* macronematous, mononematous, straight or slightly curved, cylindrical, unbranched, septate, brown or dark brown. *Conidiogenous cells* monotretic, integrated, terminal, cylindrical, brown to dark brown. *Conidia* catenate, formed in acropetal chains, straight or bent, obclavate, olivaceous to dark brown, multi-euseptate, slightly constricted at septa, distal conidia rounded at apex, truncate at base, intercalary conidia truncate at both ends, with thickened and darkened scars at base or both ends. **Sexual morph**: Undetermined.

##### Type species.

*Pleocatenatachiangraiensis* Y.R. Sun, Yong Wang bis & K.D. Hyde

##### Notes.

The morphology of *Pleocatenata* is distinguished from members in other families in Pleosporales by its tretic conidiogenous cells and catenate, euseptate conidia, and phylogenic analyses indicated it does not belong to any existing families. To avoid establishing a new family with only one species, *Pleocatenata* is introduced as a new genus and assigned to Pleosporales, genera *incertae sedis*. *Pleocatenata* is a monotypic genus reported from terrestrial habitats but without a known sexual morph. Further discovery of other species in *Pleocatenata* or phylogenetic related genera with supported monophyly will determine the familial level of *Pleocatenata*.

#### ﻿ Pleocatenata chiangraiensis

Taxon classificationFungiPleosporalesHyphomycetous

Y.R. Sun, Yong Wang bis & K.D. Hyde sp. nov.

6151EB30-06BB-56B1-A35A-7ACD4BB5427B

Index Fungorum number:IF559458

Facesoffungi number: FoF 10631

Fig. 2

##### Etymology.

The epithet referring to the location in which the fungus was collected.

##### Holotype.

MFLU: 22-0002

##### Description.

*Saprobic* on twigs of *Clerodendrumquadriloculare* and *Tarennastellulata*. **Asexual morph**: Hyphomycetous. *Colonies* on natural substrate effuse, dark, velvety. *Mycelium* immersed, composed of septate, branched, hyaline to subhyaline hyphae. *Conidiophores* macronematous, mononematous, erect, straight or slightly curved, cylindrical, unbranched, robust, 4–6-septate, brown or dark brown, rough, 35–100 µm long, 5.5–8.5 µm wide. *Conidiogenous cells* monotretic, integrated, terminal, determinate, cylindrical, dark brown. *Conidia* catenate, formed in acropetal chains of 2–3, straight or curved, obclavate, olivaceous to brown when young, blackish brown when mature, 5–8-euseptate, slightly constricted at septa, distal conidia rounded at apex, truncate at base, intercalary conidia truncate at both ends, with thickened and darkened scars at base or both ends, 34–70 µm long, 6.5–12 µm at the widest. **Sexual morph**: Unknown.

**Figure 2.**
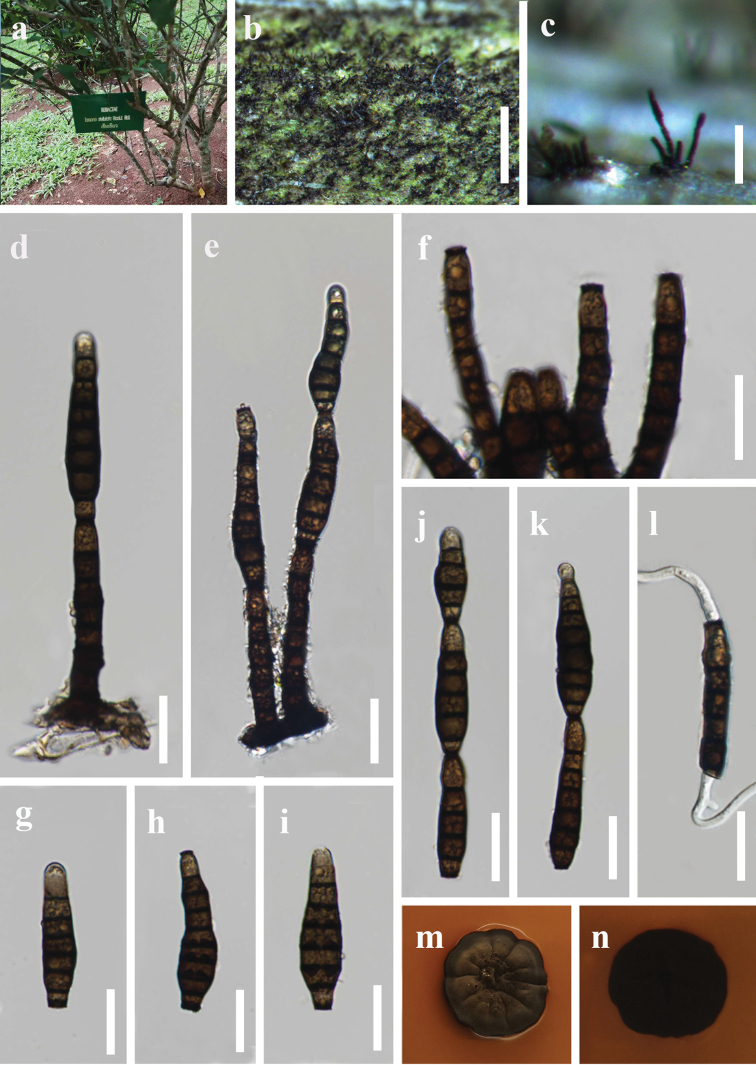
*Pleocatenatachiangraiensis* (MFLU 22-0002, holotype) **a** host (*Tarennastellulata*) **b, c** colonies on natural substrate **d, e** conidiophores with conidia **f** conidiogenous cells **g–k** conidia **l** germinated conidium **m, n** colonies on PDA (upper view and lower view). Scale bars: 1 mm (**b**); 100 μm (**c**); 20 μm (**d–l**).

##### Culture characteristics.

Conidia germinated on PDA within 12 hours at 26 °C. Germ tubes were produced from both ends. Colony reached 20–25 mm diameter after 4 weeks at room temperature on PDA media. Mycelia superficial, irregularly circular, entire edge, dark brown from above, black from below, pigment produced which turns the media reddish brown.

##### Material examined.

Thailand, Chiang Rai Province, Mae Fah Luang University, on twigs of *Tarennastellulata*, 3 July 2020, Y.R. Sun, MFU5 (MFLU 22-0002, **holotype**, ex-type living culture MFLUCC 21-0222). Thailand, Chiang Rai Province, Medicinal Plants Garden, on twigs of *Clerodendrumquadriloculare*, 7 June 2020, Y.R. Sun, B45 (MFLU 22-0001, living culture MFLUCC 21-0223).

##### Notes.

Two isolates collected from different hosts share similar morphology and clustered together in the phylogenic tree. There are no base pair differences in LSU and *tef1*-α genes between these two isolates. One base pair and two base pair differences (without gaps) are observed in ITS and *rpb2*, respectively. Therefore, the two isolates MFLUCC 21-0222 and MFLUCC 21-0223 are identified as conspecific.

## ﻿Discussion

*Pleocatenata* is phylogenetically related to Amorosiaceae, Sporormiaceae, and Sublophiostomataceae in our multi-gene analyses, but their monophyly was not well-supported, indicating their uncertain phylogenetic affinities. No hyphomycetous asexual morph has been reported in Sporormiaceae or Sublophiostomataceae (Hongsanan et al. 2020a, 2021). However, in Amorosiaceae, only two known hyphomycetous genera, *Amorosia* and *Angustimassarina*, are characterized by micronematous to semimacronematous, pale brown conidiophores, monoblastic conidiogenous cells, and single, elongate-clavate conidia (Mantle et al. 2006; Thambugala et al. 2015; Hongsanan et al. 2020a). *Pleocatenata* can be distinguished from these two genera by having monotretic conidiogenous cells and catenate, obclavate conidia.

A recently introduced species, *Corynesporasinensis* Jian Ma, X.G. Zhang & R.F. Castañeda, resembles *Pleocatenata* in its unbranched, cylindrical conidiophores and monotretic, terminal conidiogenous cells that produce catenate, obclavate conidia (Xu et al. 2020). Morphologically, *Corynesporasinensis* is more similar to *P.chiangraiensis* than to the type species of *Corynespora*, *C.cassiicola* (Berk. & M.A. Curtis) C.T. Wei (Wei 1950). Since *Corynespora* (Corynesporascaceae, Pleosporales) is a polyphyletic genus (Schoch et al. 2009; Voglmayr and Jaklitsch 2017), and there is no available sequence data for *C.sinensis*, we presume that *C.sinensis* may belong to *Pleocatenata*. However, due to lack of molecular data, and since morphology-based classification is not reliable for many hyphomycetous genera (Shenoy et al. 2006; Su et al. 2016; Yang et al. 2018), we retain the current classification. Sequences of *C.sinensis* are needed to resolve its phylogenetic placement. Detailed morphological comparison among *C.cassiicola*, *C.sinensis* and *P.chiangraiensis* is provided (Table 3).

**Table 3.** Comparison between *Corynesporacassiicola*, *C.sinensis*, and *Pleocatenatachiangraiensis*.

Species	Conidiophores	Conidiogenous cells	Conidia	References
*Corynesporacassiicola*	Unbranched, cylindrical proliferations, pale to mid brown, up to 9 septate, 110–850 × 4–11 µm	Monotretic, cylindrical, pale to mid brown	Solitary or in chains of 2–6, obclavate to cylindrical, subhyaline to pale olivaceous brown or brown, 4–20 distoseptate, 40–220 × 9–22 µm	Wei 1950
*Corynesporasinensis* (HJAUP M0156)	Unbranched, cylindrical, brown to dark, 4–8-septate, 53–96.5 × 7–8.5 µm	Monotretic, cylindrical, brown,	In chains of 2, primary conidia obclavate or fusiform, 3(–4)-distoseptate, 31.5–42 × 8–9.5 µm. secondary conidia ellipsoid, 3-distoseptate, 21–28.5 × 8–9.5 µm	Xu et al. 2020
*Pleocatenatachiangraiensis* (MFLU 21-0222)	Unbranched, cylindrical, brown or dark brown, 4–6-septate, 35–100 × 5.5–8.5 µm	Monotretic, cylindrical, dark brown	In chains of 2–3, obclavate, olivaceous to brown when young, blackish brown when mature, 5–8-euseptate, 34–70 µm × 6.5–12 µm	This study

*Pleocatenata* is similar to *Sporidesmiumsensu stricto*, which is characterized by distinctive, unbranched conidiophores, monoblastic, determinate or proliferating conidiogenous cells, and acrogenous, solitary, transversely septate conidia (Ellis 1958, 1971; Shenoy et al. 2006; Boonmee et al. 2012; Su et al. 2016; Yang et al. 2018). However, *Pleocatenata* is different from *Sporidesmium* by having catenate conidia. Additionally, *Pleocatenata* is phylogenetically distinct from *Sporidesmium*, supporting the introduction of the new genus.

The catenate, obclavate phragmoconidia of *P.chiangraiensis* are similar to capnodendron asexual morph of *Antennulariella* Woron (Antennulariellaceae, Capnodiales) (Hughes 1976, 2000; Seifert et al. 2011). Although sequence data of *Antennulariella* is not available, morphological characters, such as holoblastic conidiogenous cells and branched conidiophores of *Antennulariella*, support its separation from *P.chiangraiensis* (Hughes 1976, 2000; Seifert et al. 2011). *Pleocatenata* is also similar to *Corynesporina* Subram (Pezizomycotina, *incertae sedis*) in having unbranched, robust conidiophores and catenate conidia (Seifert et al. 2011). However, they differ in that the distoseptate conidia form in basipetal chains in *Corynesporina* and euseptate conidia form in acropetal chains in *Pleocatenata*.

## Supplementary Material

XML Treatment for Pleocatenata

XML Treatment for Pleocatenata chiangraiensis

## References

[B1] Abtahi F Nourani SL (2017) The most important fungal diseases associated with some useful medicinal plants. In: Ghorbanpour M Varma A (Eds) Medicinal plants and environmental challenges.Springer International Publishing, Cham, 279–293. 10.1007/978-3-319-68717-9_16

[B2] Abutaha N Mashaly AM Al-Mekhlafi FA Farooq M Al-shami M Wadaan MA (2015) Larvicidal activity of endophytic fungal extract of *Cochliobolusspicifer* (Pleosporales: Pleosporaceae) on *Aedescaspius* and *Culexpipiens* (Diptera: Culicidae). Applied Entomology and Zoology 50: 405–414. 10.1007/s13355-015-0347-6

[B3] Barghoorn ES (1944) Marine fungi: their taxonomy and biology. Farlowia 1: 395–467. 10.5962/p.315987

[B4] Barr ME (1987) Prodromus to class Loculoascomycetes. Amherst. University of Massachusetts, Massachusetts.

[B5] Bhagat J Kaur A Sharma M Saxena AK Chadha BS (2012) Molecular and functional characterization of endophytic fungi from traditional medicinal plants. World Journal of Microbiology and Biotechnology 28: 963–971. 10.1007/s11274-011-0894-0 22805817

[B6] Bonnardeaux Y Brundrett M Batty A Dixon K Koch J Sivasithamparam K (2007) Diversity of mycorrhizal fungi of terrestrial orchids: compatibility webs, brief encounters, lasting relationships and alien invasions. Mycological Research 111: 51–61. 10.1016/j.mycres.2006.11.006 17289365

[B7] Boonmee S Ko TWK Chukeatirote E Hyde KD Chen H Cai L McKenzie EHC Jones EBG Kodsueb R Hassan BA (2012) Two new *Kirschsteiniothelia* species with *Dendryphiopsis* anamorphs cluster in *Kirschsteiniotheliaceae* fam. nov. Mycologia 104: 698–714. 10.3852/11-089 22241611

[B8] Castañeda-Ruiz RF Heredia GP Arias RM Saikawa M Minter DW Stadler M Guarro J Decock C (2004) Two new hyphomycetes from rainforests of México, and *Briansuttonia*, a new genus to accommodate *Corynesporaalternarioides*. Mycotaxon 89: 297–305.

[B9] Chernomor O Von Haeseler A Minh BQ (2016) Terrace aware data structure for phylogenomic inference from supermatrices. Systematic Biology 65: 997–1008. 10.1093/sysbio/syw037 27121966 PMC5066062

[B10] Chomnunti P Hongsanan S Aguirre-Hudson B Tian Q Peršoh D Dhami MK Alias AS Xu JC Liu XZ Stadler M Hyde KD (2014) The sooty moulds. Fungal Diversity 66: 1–36. 10.1007/s13225-014-0278-5

[B11] Crous PW Wingfield MJ Burgess TI Hardy GESJ Barber PA Alvarado P Barnes CW Buchanan PK Heykoop M Moreno G Thangavel R van der Spuy S Barili A Barrett S Cacciola SO Cano-Lira JF Crane C Decock C Gibertoni TB Guarro J Guevara-Suarez M Hubka V Kolařík M Lira CRS Ordoñez ME Padamsee M Ryvarden L Soares AM Stchigel AM Sutton DA Vizzini A Weir BS Acharya K Aloi F Baseia IG Blanchette RA Bordallo JJ Bratek Z Butler T Cano-Canals J Carlavilla JR Chander J Cheewangkoon R Cruz RHSF da Silva M Dutta AK Ercole E Escobio V Esteve-Raventós F Flores JA Gené J Góis JS Haines L Held BW Jung MH Hosaka K Jung T Jurjević Ž Kautman V Kautmanova I Kiyashko AA Kozanek M Kubátová A Lafourcade M La Spada F Latha KPD Madrid H Malysheva EF Manimohan P Manjón JL Martín MP Mata M Merényi Z Morte A Nagy I Normand AC Paloi S Pattison N Pawłowska J Pereira OL Petterson ME Picillo B Raj KNA Roberts A Rodríguez A Rodríguez-Campo FJ Romański M Ruszkiewicz-Michalska M Scanu B Schena L Semelbauer M Sharma R Shouche YS Silva V Staniaszek-Kik M Stielow JB Tapia C Taylor PWJ Toome-Heller M Vabeikhokhei JMC van Diepeningen AD Van Hoa N M VT Wiederhold NP Wrzosek M Zothanzama J Groenewald JZ (2017) Fungal Planet description sheets: 558–624. Persoonia 38: 240–384. 10.3767/003158517X698941 29151634 PMC5645186

[B12] Ellis MB (1958) *Clasterosporium* and some allied Dematiaceae Phragmosporae: I. Mycological Papers 7: 1–89.

[B13] Ellis MB (1971) Dematiaceous hyphomycetes. Commonwealth Mycological Institute, Kew.

[B14] Ferdinandez HS Manamgoda DS Udayanga D Deshappriya N Munasinghe MS Castlebury LA (2021) Molecular phylogeny and morphology reveal three novel species of *Curvularia* (Pleosporales, Pleosporaceae) associated withcereal crops and weedy grass hosts. Mycological Progress 20: 431–451. 10.1007/s11557-021-01681-0

[B15] Hall TA (1999) BioEdit: a user-friendly biological sequence alignment editor and analysis program for Windows 95/98/NT. In, 95–98.

[B16] Hashimoto A Matsumura M Hirayama K Tanaka K (2017) Revision of *Lophiotremataceae* (*Pleosporales*, *Dothideomycetes*): *Aquasubmersaceae*, *Cryptocoryneaceae*, and *Hermatomycetaceae* fam. nov. Persoonia 39: 51–73. 10.3767/persoonia.2017.39.03 29503470 PMC5832957

[B17] Hoang DT Chernomor O Von Haeseler A Minh BQ Vinh LS (2018) UFBoot2: improving the ultrafast bootstrap approximation. Molecular Biology and Evolution 35: 518–522. 10.1093/molbev/msx281 29077904 PMC5850222

[B18] Hongsanan S Hyde KD Phookamsak R Wanasinghe DN McKenzie EHC Sarma VV Boonmee S Lücking R Bhat DJ Liu NG Tennakoon DS Pem D Karunarathna A Jiang SH Jones EBG Phillips AJL Manawasinghe IS Tibpromma S Jayasiri SC Sandamali DS Jayawardena RS Wijayawardene NN Ekanayaka AH Jeewon R Lu YZ Dissanayake AJ Zeng XY Luo ZL Tian Q Phukhamsakda C Thambugala KM Dai DQ Chethana KWT Samarakoon MC Ertz D Bao DF Doilom M Liu JK Pérez-Ortega S Suija A Senwanna C Wijesinghe SN Konta S Niranjan M Zhang SN Ariyawansa HA Jiang HB Zhang JF Norphanphoun C de Silva NI Thiyagaraja V Zhang H Bezerra JDP Miranda-González R Aptroot A Kashiwadani H Harishchandra D Sérusiaux E Aluthmuhandiram JVS Abeywickrama PD Devadatha B Wu HX Moon KH Gueidan C Schumm F Bundhun D Mapook A Monkai J Chomnunti P Suetrong S Chaiwan N Dayarathne MC Yang J Rathnayaka AR Bhunjun CS Xu JC Zheng JS Liu G Feng Y Xie N (2020a) Refined families of Dothideomycetes: Dothideomycetidae and Pleosporomycetidae. Mycosphere 11: 1553–2107. 10.5943/mycosphere/11/1/13

[B19] Hongsanan S Hyde KD Phookamsak R Wanasinghe DN McKenzie EHC Sarma VV Lücking R Boonmee S Bhat JD Liu NG Tennakoon DS Pem D Karunarathna A Jiang SH Jones GEB Phillips AJL Manawasinghe IS Tibpromma S Jayasiri SC Sandamali D Jayawardena RS Wijayawardene NN Ekanayaka AH Jeewon R Lu YZ Phukhamsakda C Dissanayake AJ Zeng XY Luo ZL Tian Q Thambugala KM Dai D Samarakoon MC Chethana KWT Ertz D Doilom M Liu JK Pérez-Ortega S Suija A Senwanna C Wijesinghe SN Niranjan M Zhang SN Ariyawansa HA Jiang HB Zhang J-F Norphanphoun C de Silva NI Thiyagaraja V Zhang H Bezerra JDP Miranda-González R Aptroot A Kashiwadani H Harishchandra D Sérusiaux E Abeywickrama PD Bao D-F Devadatha B Wu HX Moon KH Gueidan C Schumm F Bundhun D Mapook A Monkai J Bhunjun CS Chomnunti P Suetrong S Chaiwan N Dayarathne MC Yang J Rathnayaka AR Xu JC Zheng J Liu G Feng Y Xie N (2020b) Refined families of Dothideomycetes: orders and families incertae sedis in Dothideomycetes. Fungal Diversity 105: 17–318. 10.1007/s13225-020-00462-6

[B20] Hongsanan S Phookamsak R Goonasekara ID Thambugala KM Hyde KD Bhat JD Suwannarach N Cheewangkoon R (2021) Introducing a new pleosporalean family Sublophiostomataceae fam. nov. to accommodate *Sublophiostoma* gen. nov. Scientific Reports 11: e9496. 10.1038/s41598-021-88772-w PMC8096836 33947898

[B21] Huang WY Cai YZ Hyde KD Corke H Sun M (2008) Biodiversity of endophytic fungi associated with 29 traditional Chinese medicinal plants. Fungal Diversity 33: 61–75.

[B22] Hughes SJ (1976) Sooty moulds. Mycologia 68: 693–820. 10.2307/3758799

[B23] Hughes SJ (2000) *Antennulariellabatistae* n. sp. and its *Capnodendron* and *Antennariella* synanamorphs, with notes on *Capnodiumcapsuliferum*. Canadian Journal of Botany 78: 1215–1226. 10.1139/b00-098

[B24] Hyde KD Jones EBG Liu JK Ariyawansa H Boehm E Boonmee S Braun U Chomnunti P Crous PW Dai DQ Diederich P Dissanayake A Doilom M Doveri F Hongsanan S Jayawardena R Lawrey JD Li YM Liu YX Lücking R Monkai J Muggia L Nelsen MP Pang KL Phookamsak R Senanayake IC Shearer CA Suetrong S Tanaka K Thambugala KM Wijayawardene NN Wikee S Wu HX Zhang Y Aguirre-Hudson B Alias SA Aptroot A Bahkali AH Bezerra JL Bhat DJ Camporesi E Chukeatirote E Gueidan C Hawksworth DL Hirayama K De Hoog S Kang JC Knudsen K Li WJ Li XH Liu ZY Mapook A McKenzie EHC Miller AN Mortimer PE Phillips AJL Raja HA Scheuer C Schumm F Taylor JE Tian Q Tibpromma S Wanasinghe DN Wang Y Xu JC Yacharoen S Yan JY Zhang M (2013) Families of Dothideomycetes. Fungal Diversity 63: 1–313. 10.1007/s13225-013-0263-4

[B25] Hyde KD Chaiwan N Norphanphoun C Boonmee S Camporesi E Chethana KWT Dayarathne MC de Silva NI Dissanayake AJ Ekanayaka AH Hongsanan S Huang SK Jayasiri SC Jayawardena RS Jiang HB Karunarathna A Lin CG Liu JK Liu NG Lu YZ Luo ZL Maharachchimbura SSN Manawasinghe IS Pem D Perera RH Phukhamsakda C Samarakoon MC Senwanna C Shang QJ Tennakoon DS Thambugala KM Tibpromma S Wanasinghe DN Xiao YP Yang J Zeng XY Zhang JF Zhang SN Bulgakov TS Bhat DJ Cheewangkoon R Goh TK Jones EBG Kang JC Jeewon R Liu ZY Lumyong S Kuo CH McKenzie EHC Wen TC Yan JY Zhao Q (2018) Mycosphere notes 169–224. Mycosphere 9: 271–430. 10.5943/mycosphere/9/2/8

[B26] Hyde KD Xu JC Rapior S Jeewon R Lumyong S Niego AGT Abeywickrama PD Aluthmuhandiram JVS Brahamanage RS Brooks S Chaiyasen A Chethana KWT Chomnunti P Chepkirui C Chuankid B de Silva NI Doilom M Faulds C Gentekaki E Gopalan V Kakumyan P Harishchandra D Hemachandran H Hongsanan S Karunarathna A Karunarathna SC Khan S Kumla J Jayawardena RS Liu JK Liu NG Luangharn T Macabeo APG Marasinghe DS Meeks D Mortimer PE Mueller P Nadir S Nataraja KN Nontachaiyapoom S O’Brien M Penkhrue W Phukhamsakda C Ramanan US Rathnayaka AR Sadaba RB Sandargo B Samarakoon BC Tennakoon DS Siva R Sriprom W Suryanarayanan TS Sujarit K Suwannarach N Suwunwong T Thongbai B Thongklang N Wei D Wijesinghe SN Winiski J Yan J Yasanthika E Stadler M (2019) The amazing potential of fungi: 50 ways we can exploit fungi industrially. Fungal Diversity 97: 1–136. 10.1007/s13225-019-00430-9

[B27] Hyde KD Dong Y Phookamsak R Jeewon R Bhat DJ Jones EBG Liu NG Abeywickrama PD Mapook A Wei DP Perera RH Manawasinghe IS Pem D Bundhun D Karunarathna A Ekanayaka AH Bao DF Li JF Samarakoon MC Chaiwan N Lin CG Phutthacharoen K Zhang SN Senanayake IC Goonasekara ID Thambugala KM Phukhamsakda C Tennakoon DS Jiang HB Yang J Zeng M Huanraluek N Liu JK Wijesinghe SN Tian Q Tibpromma S Brahmanage RS Boonmee S Huang SK Thiyagaraja V Lu YZ Jayawardena RS Dong W Yang EF Singh SK Singh SM Rana S Lad SS Anand G Devadatha B Niranjan M Sarma VV Liimatainen K Aguirre-Hudson B Niskanen T Overall A Alvarenga RLM Gibertoni TB Pfliegler WP Horváth E Imre A Alves AL da Silva Santos AC Tiago PV Bulgakov TS Wanasinghe DN Bahkali AH Doilom M Elgorban AM Maharachchikumbura SSN Rajeshkumar KC Haelewaters D Mortimer PE Zhao Q Lumyong S Xu J Sheng J (2020) Fungal diversity notes 1151–1276: taxonomic and phylogenetic contributions on genera and species of fungal taxa. Fungal Diversity 100: 5–277. 10.1007/s13225-020-00439-5

[B28] Iturrieta‐González I Pujol I Iftimie S García D Morente V Queralt R Guevara-Suarez M Alastruey‐Izquierdo A Ballester F Hernández-Restrepo M (2020) Polyphasic identification of three new species in AlternariasectionInfectoriae causing human cutaneous infection. Mycoses 63: 212–224. 10.1111/myc.13026 31651065

[B29] Jayasiri SC Hyde KD Ariyawansa HA Bhat J Buyck B Cai L Dai YC Abd-Elsalam KA Ertz D Hidayat I Jeewon R Jones EBG Bahkali AH Karunarathna SC Liu JK Luangsa-ard JJ Lumbsch HT Maharachchikumbura SSN McKenzie EHC Moncalvo JM Ghobad-Nejhad M Nilsson H Pang KL Pereira OL Phillips AJL Raspé O Rollins AW Romero AI Etayo J Selçuk F Stephenson SL Suetrong S Taylor JE Tsui CKM Vizzini A Abdel-Wahab MA Wen TC Boonmee S Dai DQ Daranagama DA Dissanayake AJ Ekanayaka AH Fryar SC Hongsanan S Jayawardena RS Li WJ Perera RH Phookamsak R de Silva NI Thambugala KM Tian Q Wijayawardene NN Zhao RL Zhao Q Kang JC Promputtha I (2015) The Faces of Fungi database: fungal names linked with morphology, phylogeny and human impacts. Fungal Diversity 74: 3–18. 10.1007/s13225-015-0351-8

[B30] Kirk PM Cannon PF Minter DW Staplers JA (2008) Dictionary of the Fungi 10^th^ edn. CABI Bioscience, UK.

[B31] Larsson A (2014) AliView: a fast and lightweight alignment viewer and editor for large datasets. Bioinformatics 30: 3276–3278. 10.1093/bioinformatics/btu531 25095880 PMC4221126

[B32] Li GJ Hyde KD Zhao RL Hongsanan S Abdel-Aziz FA Abdel-Wahab MA Alvarado P Alves-Silva G Ammirati JF Ariyawansa HA Baghela A Bahkali AH Beug M Bhat DJ Bojantchev D Boonpratuang T Bulgakov TS Camporesi E Boro MC Ceska O Chakraborty D Chen JJ Chethana KWT Chomnunti P Consiglio G Cui BK Dai DQ Dai YC Daranagama DA Das K Dayarathne MC De Crop E De Oliveira RJV de Souza CAF de Souza JI Dentinger BTM Dissanayake AJ Doilom M Drechsler-Santos ER Ghobad-Nejhad M Gilmore SP Góes-Neto A (2016) Fungal diversity notes 253–366: taxonomic and phylogenetic contributions to fungal taxa. Fungal Diversity 78: 1–237. 10.1007/s13225-016-0366-9

[B33] Li H Sun G Batzer JC Crous PW Groenewald JZ Karakaya A Gleason ML (2011) *Scleroramularia* gen. nov. associated with sooty blotch and flyspeck of apple and pawpaw from the Northern Hemisphere. Fungal Diversity 46: 53–66. 10.1007/s13225-010-0074-9

[B34] Liu YJ Whelen S Hall BD (1999) Phylogenetic relationships among Ascomycetes: evidence from an RNA polymerse II subunit. Molecular Biology and Evolution 16: 1799–1808. 10.1093/oxfordjournals.molbev.a026092 10605121

[B35] Long H Zhang Q Hao YY Shao XQ Wei XX Hyde KD Wang Y Zhao DG (2019) *Diaporthe* species in south-western China. MycoKeys 57: 113–127. 10.3897/mycokeys.57.35448 31523165 PMC6717119

[B36] Luttrell ES (1955) The ascostromatci Ascomycetes. Mycologia 47: 511–532. 10.2307/3755666

[B37] Ma XY Maharachchikumbura SSN Chen BW Hyde KD McKenzie EHC Chomnunti P Kang JC (2019) Endophytic pestalotiod taxa in *Dendrobiumorchids*. Phytotaxa 419: 268–286. 10.11646/phytotaxa.419.3.2

[B38] Mantle PG Hawksworth DL Pazoutova S Collinson LM Rassing BR (2006) *Amorosialittoralis* gen. sp. nov., a new genus and species name for the scorpinone and caffeine-producing hyphomycete from the littoral zone in The Bahamas. Mycological Research 110: 1371–1378. 10.1016/j.mycres.2006.09.013 17101270

[B39] Mathiyazhagan S Kavitha K Nakkeeran S Chandrasekar G Manian K Renukadevi P Krishnamoorthy AS Fernando WGD (2004) PGPR mediated management of stem blight of *Phyllanthusamarus* (Schum and Thonn) caused by *Corynesporacassiicola* (Berk and Curt) Wei. Archives of Phytopathology and Plant Protection 37: 183–199. 10.1080/03235400410001730658

[B40] Miller MA Pfeiffer W Schwartz T (2010) “Creating the CIPRES Science Gateway for inference of large phylogenetic trees” in Proceedings of the Gateway Computing Environments Workshop (GCE), 14 Nov. 2010, New Orleans, LA, 1–8. 10.1109/GCE.2010.5676129

[B41] Minh BQ Nguyen MAT von Haeseler A (2013) Ultrafast approximation for phylogenetic bootstrap. Molecular Biology and Evolution 30: 1188–1195. 10.1093/molbev/mst024 23418397 PMC3670741

[B42] Nguyen LT Schmidt HA Von Haeseler A Minh BQ (2015) IQ-TREE: a fast and effective stochastic algorithm for estimating maximum-likelihood phylogenies. Molecular Biology and Evolution 32: 268–274. 10.1093/molbev/msu300 25371430 PMC4271533

[B43] Nylander JAA (2004) MrModeltest v2.2. Program distributed by the author: 2. Evolutionary Biology Centre, Uppsala University, 1–2.

[B44] Obrist W (1959) Untersuchungen über einige” dothideale” Gattungen. Phytopathologische Zeitschrift 35: 357–388. 10.1111/j.1439-0434.1959.tb01833.x

[B45] Ramesh C (2003) *Loculoascomycetes* from India. Rao GP, Manoharachari C, Bhat DJ (Eds) Frontiers of Fungal Diversity in India, International Book Distributing Company, Lucknow, India, 457–479.

[B46] Rambaut A Drummond A (2008) FigTree: Tree figure drawing tool, version 1.2. 2. Institute of Evolutionary Biology, University of Edinburgh.

[B47] Rannala B Yang ZH (1996) Probability distribution of molecular evolutionary trees: A new method of phylogenetic inference. Journal of Molecular Evolution 43: 304–311. 10.1007/BF02338839 8703097

[B48] Rasool-Hassan BA (2012) Medicinal plants (importance and uses). Pharmaceut Anal Acta 3: 2153–2435. 10.4172/2153-2435.1000e139

[B49] Rehner SA Samuels GJ (1994) Taxonomy and phylogeny of *Gliocladium* analysed from nuclear large subunit ribosomal DNA sequences. Mycological Research 98: 625–634. 10.1016/S0953-7562(09)80409-7

[B50] Rehner SA Buckley E (2005) A beauveria phylogeny inferred from nuclear ITS and EF1-α sequences: evidence for cryptic diversification and links to *Cordycepsteleomorphs*. Mycologia 97(1): 84–98. 10.1080/15572536.2006.11832842 16389960

[B51] Ronquist F Teslenko M Van Der Mark P Ayres DL Darling A Höhna S Larget B Liu L Suchard MA Huelsenbeck JP (2012) MrBayes 3.2: efficient Bayesian phylogenetic inference and model choice across a large model space. Systematic Biology 61: 539–542. 10.1093/sysbio/sys029 22357727 PMC3329765

[B52] Schoch C Crous PW Groenewald JZ Boehm E Burgess TI De Gruyter J De Hoog GS Dixon L Grube M Gueidan C (2009) A class-wide phylogenetic assessment of Dothideomycetes. Studies in Mycology 64: 1–15. 10.3114/sim.2008.61.08 20169021 PMC2816964

[B53] Seifert K Morgan-Jones G Gams W Kendrick B (2011) The genera of hyphomycetes. CBS–KNAW Fungal Biodiversity Centre, Utrecht.

[B54] Senanayake IC Rathnayake AR Marasinghe DS Calabon MS Gentekaki E Lee HB Hurdeal VG Pem D Dissanayake LS Wijesinghe SN Bundhun D Nguyen TT Goonasekara ID Abeywickrama PD Bhunjun CS Jayawardena RS Wanasinghe DN Jeewon R Bhat DJ Xiang MM (2020) Morphological approaches in studying fungi: collection, examination, isolation, sporulation and preservation. Mycosphere 11: 2678–2754. 10.5943/mycosphere/11/1/20

[B55] Shenoy BD Jeewon R Wu WP Bhat DJ Hyde KD (2006) Ribosomal and *RPB2* DNA sequence analyses suggest that *Sporidesmium* and morphologically similar genera are polyphyletic. Mycological Research 110: 916–928. 10.1016/j.mycres.2006.06.004 16908125

[B56] Strobel G Stierle A Stierle D Hess WM (1993) *Taxomycesandreanae*, a proposed new taxon for a bulbilliferous hyphomycete associated with Pacific Yew (*Taxusbrevifolia*). Mycotaxon. 47: 71–80.

[B57] Su H Kang JC Cao JJ Mo L Hyde KD (2014) Medicinal plant endophytes produce analogous bioactive compounds. Chiang Mai Journal Science 41: 1–13.

[B58] Su HY Hyde KD Maharachchikumbura SSN Ariyawansa HA Luo ZL Promputtha I Tian Q Lin CG Shang QJ Zhao YC Chai HM Liu XY Bahkali AH Bhat JD McKenzie EHC Zhou DQ (2016) The families *Distoseptisporaceae* fam. nov., *Kirschsteiniotheliaceae*, *Sporormiaceae* and *Torulaceae*, with new species from freshwater in Yunnan Province, China. Fungal Diversity 80: 375–409. 10.1007/s13225-016-0362-0

[B59] Sun JZ Liu XZ McKenzie EHC Jeewon R Liu JK Zhang XL Zhao Q Hyde KD (2019) Fungicolous fungi: terminology, diversity, distribution, evolution, and species checklist. Fungal Diversity 95: 337–430. 10.1007/s13225-019-00422-9

[B60] Sun YR Jayawardena RS Hyde KD Wang Y (2021) *Kirschsteiniotheliathailandica* sp. nov. (Kirschsteiniotheliaceae) from Thailand. Phytotaxa 490(2): 172–182. 10.11646/phytotaxa.490.2.3

[B61] Tan YP Crous PW Shivas RG (2016) Eight novel *Bipolaris* species identified from John L. Alcorn’s collections at the Queensland Plant Pathology Herbarium (BRIP). Mycological Progress 15: 1203–1214. 10.1007/s11557-016-1240-6

[B62] Tennakoon DS Kuo CH Maharachchikumbura SSN Thambugala KM Gentekaki E Phillips AJL Bhat DJ Wanasinghe DN de Silva NI Promputtha I Hyde KD (2021) Taxonomic and phylogenetic contributions to *Celtisformosana*, *Ficusampelas*, *F.septica*, *Macarangatanarius* and *Morusaustralis* leaf litter inhabiting microfungi. Fungal Diversity 108: 1–215. 10.1007/s13225-021-00474-w

[B63] Thambugala KM Hyde KD Tanaka K Tian Q Wanasinghe DN Ariyawansa HA Jayasiri SC Boonmee S Camporesi E Hashimoto A Hirayama K Schumacher RK Promputtha I Liu ZY (2015) Towards a natural classification and backbone tree for Lophiostomataceae, Floricolaceae, and Amorosiaceae fam. nov. Fungal Diversity 74: 199–266. 10.1007/s13225-015-0348-3

[B64] Tóth S (1975) Some new microscopic fungi, III. Annales Historico-naturales Musel nationalis Hungarici 67: 31–35.

[B65] Trifinopoulos J Nguyen LT von Haeseler A Minh BQ (2016) W-IQ-TREE: a fast online phylogenetic tool for maximum likelihood analysis. Nucleic Acids Research 44: W232–W235. 10.1093/nar/gkw256 PMC4987875 27084950

[B66] Tzean SS Chen JL (1990) *Cheiromoniliophoraelegans* gen. et sp. nov. (Hyphomycetes). Mycological Research 94: 424–427. 10.1016/S0953-7562(09)80373-0

[B67] Vaidya G Lohman DJ Meier R (2011) SequenceMatrix: concatenation softwarefor the fast assembly of multi‐gene datasets with character set and codon information. Cladistics 27: 171–180. 10.1111/j.1096-0031.2010.00329.x 34875773

[B68] Vilgalys R Hester M (1990) Rapid genetic identification and mapping of enzymatically amplified ribosomal DNA from several *Cryptococcus* species. Journal of Bacteriology 172: 4238–4246. 10.1128/jb.172.8.4238-4246.1990 2376561 PMC213247

[B69] Voglmayr H Jaklitsch WM (2017) *Corynespora*, *Exosporium* and *Helminthosporium* revisited – New species and generic reclassification. Studies in Mycology 87: 43–76. 10.1016/j.simyco.2017.05.001 28649153 PMC5473648

[B70] Wei CT (1950) Notes on *Corynespora*. Mycological Papers 34, 10 pp.

[B71] White TJ Bruns T Lee SJWT Taylor J (1990) Amplification and direct sequencing of fungal ribosomal RNA genes for phylogenetics. In: Innis M Gelfand D Shinsky J White T (Eds) PCR protocols: a guide to methods and applications.Academic Press, New York, 315–322. 10.1016/B978-0-12-372180-8.50042-1

[B72] Wijayawardene NN Crous PW Kirk PM Hawksworth DL Boonmee S Braun U Dai DQ D’souza MJ Diederich P Dissanayake A Doilom M Hongsanan S Jones EBG Groenewald JZ Jayawardena R Lawrey JD Liu JK Lücking R Madrid H Manamgoda DS Muggia L Nelsen MP Phookamsak R Suetrong S Tanaka K Thambugala KM Wanasinghe DN Wikee S Zhang Y Aptroot A Ariyawansa HA Bahkali AH Bhat DJ Gueidan C Chomnunti P De Hoog GS Knudsen K Li WJ McKenzie EHC Miller AN Phillips AJL Piątek M Raja HA Shivas RS Slippers B Taylor JE Tian Q Wang Y Woudenberg JHC Cai L Jaklitsch WM Hyde KD (2014) Naming and outline of Dothideomycetes–2014 including proposals for the protection or suppression of generic names. Fungal Diversity 69: 1–55. 10.1007/s13225-014-0309-2 27284275 PMC4896388

[B73] Wijayawardene NN Hyde KD Al-Ani LKT Tedersoo L Haelewaters D Rajeshkumar KC Zhao RL Aptroot A Leontyev D Saxena RK Tokarev YS Dai DQ Letcher PM Stephenson SL Ertz D Lumbsch HT Kukwa M Issi IV Madrid H Phillips AJL Selbmann L Pfliegler WP Horváth E Bensch K Kirk PM Kolaříková K Raja HA Radek R Papp V Dima V Ma J Malosso E Takamatsu S Rambold G Gannibal PB Triebel D Gautam AK Avasthi S Suetrong S Timdal E Fryar SC Delgado G Réblová M Doilom M Dolatabadi S Pawłowska JZ Humber RA Kodsueb R Sánchez-Castro I Goto BT Silva DKA de Souza FA Oehl F da Silva GA Silva IR Błaszkowski J Jobim K Maia LC Barbosa FR Fiuza PO Divakar PK Shenoy BD Castañeda-Ruiz RF Somrithipol S Lateef AA Karunarathna SC Tibpromma S Mortimer PE Wanasinghe DN Phookamsak R Xu J Wang Y Tian F Alvarado P Li DW Kušan I Matočec N Mešić A Tkalčec Z Maharachchikumbura SSN Papizadeh M Heredia G Wartchow F Bakhshi M Boehm E Youssef N Hustad VP Lawrey JD Santiago ALCMA Bezerra JDP Souza-Motta CM Firmino AL Tian Q Houbraken J Hongsanan S Tanaka K Dissanayake AJ Monteiro JS Grossart HP Suija A Weerakoon G Etayo J Tsurykau A Vázquez V Mungai P Damm U Li QR Zhang H Boonmee S Lu YZ Becerra AG Kendrick B Brearley FQ Motiejūnaitė J Sharma B Khare R Gaikwad S Wijesundara DSA Tang LZ He MQ Flakus A Rodriguez-Flakus P Zhurbenko MP McKenzie EHC Stadler M Bhat DJ Liu JK Raza M Jeewon R Nassonova ES Prieto M Jayalal RGU Erdoğdu M Yurkov A Schnittler M Shchepin ON Novozhilov YK Silva-Filho AGS Gentekaki E Liu P Cavender JC Kang Y Mohammad S Zhang LF Xu RF Li YM Dayarathne MC Ekanayaka AH Wen TC Deng CY Pereira OL Navathe S Hawksworth DL Fan XL Dissanayake LS Kuhnert E Grossart HP Thines M (2020) Outline of Fungi and fungus-like taxa. Mycosphere 11: 1060–1456. 10.5943/mycosphere/11/1/8

[B74] Wijayawardene NN Hyde KD Anand G Dissanayake LS Tang LZ Dai DQ (2021) Towards incorporating asexually reproducing fungi in the natural classification and notes for pleomorphic genera. Mycosphere 12: 238–405. 10.5943/mycosphere/12/1/4

[B75] Xu ZH Kuang WG Qiu L Zhang XG Castañeda-Ruíz RF Ma J (2020) *Corynesporasinensis* sp. nov. from Jiangxi, China. Mycotaxon 135: 803–809. 10.5248/135.803

[B76] Yang J Maharachchikumbura SSN Liu JK Hyde KD Jones EBG Al-Sadi AM Liu ZY (2018) *Pseudostanjehughesiaaquitropica* gen. et sp. nov. and *Sporidesmium**sensu lato* species from freshwater habitats. Mycological Progress 17: 591–616. 10.1007/s11557-017-1339-4

[B77] Zhang Q Yang ZF Cheng W Wijayawardene NN Hyde KD Chen Z Wang Y (2020) Diseases of *Cymbopogoncitratus* (Poaceae) in China: *Curvulariananningensis* sp. nov. MycoKeys 63: 49–67. 10.3897/mycokeys.63.49264 32099520 PMC7033261

[B78] Zhang SN Hyde KD Gareth Jones EB Cheewangkoon R Liu JK (2018) *Acuminatisporapalmarum* gen. et sp. nov. from mangrove habitats. Mycological Progress 17: 1173–1188. 10.1007/s11557-018-1433-2

[B79] Zhang Y Crous PW Schoch CL Hyde KD (2012) Pleosporales. Fungal Diversity 53: 1–221. 10.1007/s13225-011-0117-x 23097638 PMC3477819

